# Preparation of a self-matting, anti-fingerprint and skin-tactile wood coating via biomimetic self-wrinkling patterns

**DOI:** 10.1038/s41598-024-64385-x

**Published:** 2024-06-10

**Authors:** Yingchun Sun, Ru Liu, Ling Long, Yuhui Sun

**Affiliations:** Research Institute of Wood Industry, Chinese Academy of Forestry, Beijing, 100091 China

**Keywords:** PUA, Wood coating, Biomimetic structure, Photopolymerization, Functional performance, Lasers, LEDs and light sources, Optical materials and structures, Polymer chemistry

## Abstract

Inspired by natural wrinkled surfaces, artificial surfaces with biomimetic wrinkled structures have been widely used to improve optical properties, wettability, and antibacterial properties. However, the preparation of wrinkled structures has the disadvantages of long-time consumption and complex processes. Herein, we prepared a self-wrinkling polyurethane-acrylate (PUA) wood coating via biomimetic self-wrinkling patterns by using a light-emitting diode (LED)/excimer/mercury lamp curing system, which was capable of self-matting, anti-fingerprint and skin-tactile performance. By adjusting the irradiation intensity in the curing system, the wavelength (λ) and amplitude (A) of wrinkles on the coating surface were controlled to enhance the coating performance. After curing by the LED, excimer, and mercury lamps at energy intensities of 500, 30, and 300 mW/cm^2^ respectively, the self-wrinkling coating showed excellent surface performance. The self-wrinkling coating represented low gloss of 4.1 GU at 85°, high hardness of 4H. Interestingly, the coating surface had a high hydrophobicity (104.5°) and low surface energy (29–30 mN/m) and low coefficient (COF) of friction (0.1–0.2), which were consistent with those of the human skin surface. Besides, the wrinkled structure also improved the thermal stability of the coating samples. This study provided a promising technique for the mass production of self-wrinkling coatings that could be used in wood-based panels, furniture, and leather.

## Introduction

Wrinkled surfaces are ubiquitous in nature, with the best example being biological surfaces such as lotus leaves, insect epidermis, and human skin^1–5^. Owing to their unique surface structures, wrinkled surfaces typically exhibit excellent light reflectivity, wettability, and adhesion. Consequently, artificial surfaces with biomimetic wrinkled structures are being extensively applied in fields like optics, self-cleaning coatings, and flexible electronics^6–10^, and it has become important to develop a simple and efficient method for fabricating self-wrinkling coatings.

As a common phenomenon in nature, wrinkle formation of wrinkles is generally spontaneous^11–12^. Compared to the wrinkles formed under external pressure and temperature, self-wrinkling coatings that are formed spontaneously can save on costs and time. Photopolymerization is widely used as an accurate, convenient, and fast method for preparing self-wrinkling coatings. The coatings formed by this process, which mainly involves UV light irradiation, are also known as photopolymerization self-wrinkling coatings. Researchers have found that by altering the coating thickness^13^, photoinitiator content, crosslinking agent^14^, oxygen concentration, and irradiation time^15^, the wavelength and amplitude of wrinkles on the coating surface can be regulated. Based on these results, preparing self-wrinkling coatings by simulating wrinkled surfaces observed in nature via photopolymerization is a promising direction.

Recently, wrinkling of surface structures has proven to be an effective method to generate functionalized surfaces. Therefore, a series of self-wrinkling coatings have been prepared via photopolymerization and applied in the fields of electronic and optical devices. For instance, Gao et al. utilized photomask aggregation to generate the hierarchical patterns of self-wrinkling coatings, which can be used in anti-counterfeiting and packaging of light-emitting diode (LED) chips^16^. Similarly, Chen et al. used biodegradable polymers to prepare a frog skin-inspired self-wrinkling coating, which has good antibacterial and anti-fouling properties^17^. Cheng et al. prepared a radiation cooling coating comprising a biomimetic structure similar to that of natural wrinkles on the human skin, and this coating increased the energy-saving rate of air conditioning to 50%^18^. However, the current preparation process used for self-wrinkling coatings shows low applicability since it does not provide sufficient controllability over surface wrinkles and coating roughness. Thus, it is necessary to develop a simple, controllable, and effective method for preparing large-scale self-wrinkling coatings that can regulate the wavelength and amplitude of wrinkles as well as the coating roughness.

**Figure 1:**
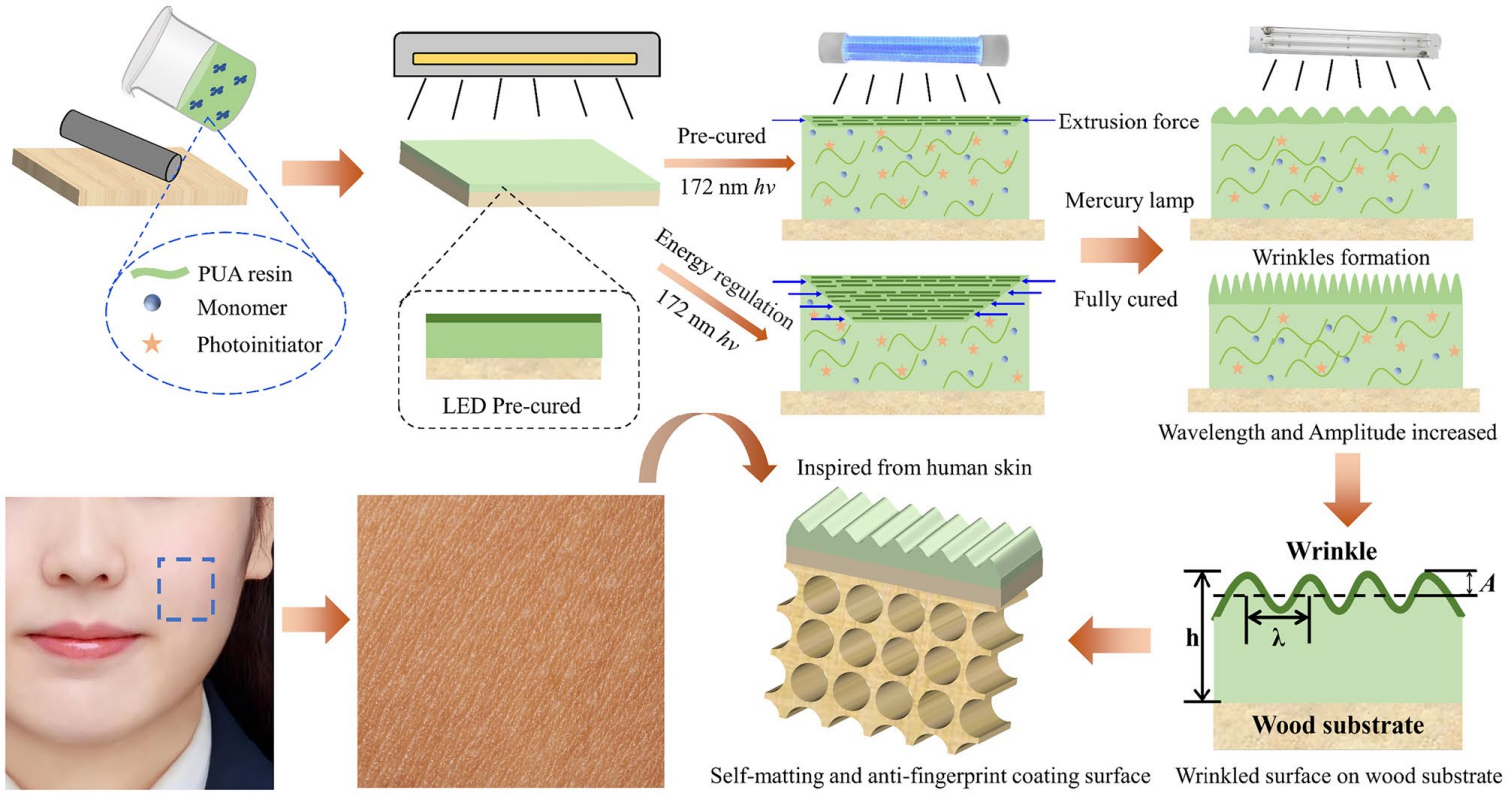
The preparation and fabricated strategy of self-wrinkling PUA coating.

Herein we reported a strategy to prepare biomimetic self-wrinkling patterns on wood surface through a light-emitting diode (LED)/excimer/mercury lamp curing system, thereby imparting excellent self-matting, anti-fingerprint and mechanical performances during practical application. Inspired by the wrinkled structure of human skin, the self-wrinkling structure of the wood surface was achieved by using the difference in penetration depth of different UV light to create modulus differences and horizontal shrinkage stresses through a gradient curing system (Fig. 1). There were literatures report that influences factors during the curing process could change the wrinkled structure of the coating surface. However, to the best of our knowledge, irradiation energy as a key variable had not been studied. Thus, to further regulate the wavelength and amplitude of the coating surface, the coating samples were treated by varying the irradiation energy to obtain the required surface properties of the wood product. The surface structure and chemical reactions were characterized, the surface performances including optics, wettability, mechanics and durability were tested. Furthermore, the morphology, roughness, and friction coefficient of the self-wrinkling coating surface were similar to the human skin, and exhibited skin-tactile performance on the wood surface. This study presents an effective strategy for the mass production of high-performance wood products with a good feeling in touch, which can be used in wood-based panels, furniture, and leather.

## Experimental

### Materials

The monomers (trimethylolpropane triacrylate, TMPTA; 1,3-butanediol diacrylate, BDDA), oligomers (2-hydroxyethyl acrylate, HEA; PU_3_, triisocyanate based polyurethane), benzophenone (BP) and Methyl benzoylformate (MBF) photoinitiator with analytical reagent (AR) level were purchased from Shanghai Hechuang Chemical Technology Co. Ltd., Shanghai, China. The birch plywood with dimensions of 900 mm × 150 mm × 18 mm (thickness) was provided by Shenghuo Home Collection Co. Ltd., China. The distilled water used in this study was prepared in the laboratory. All the materials were utilized as received without purification.

### Preparation of self-wrinkling PUA coating

The mixture of UV-PUA coating was roll-coated to form a thin film on the birch plywood. The Prior to roll coating, the birch plywood was treated with one layer of putty and three layers of UV primer coatings (epoxy primer). The amount of wet coating was about 15 g/m^2^, the line speed of the coating roll was 10 m/min, and the coating thickness was about 60 μm. The resulting wet coating was exposed to LED lamp (365 nm), excimer lamp (172 nm), and UV mercury (300–600 nm) lamp (PRT-C1103a, PRT-L2 and PRT-1320, Foshan Shunde PURETE Mechanical Co., Ltd.). The coating samples were treated with different energy intensities for LED lamp (0, 200, 500, 800, and 1000 mW/cm^2^), excimer lamp (0, 10, 20, 30, and 40 mW/cm^2^), and UV mercury lamp (150, 225, 300 mW/cm^2^) for 10 s (Fig. 1). Meanwhile, the control PUA coating samples were cured by mercury lamp at 300 mW/cm^2^ energy intensity. The self-wrinkling coating samples prepared by the 172 nm excimer lamp was protecting in the nitrogen of 99.99% concentration. The preparation details for self-wrinkling PUA coating samples are shown in Table 1.

**Table 1:** Preparation method used for self-wrinkling PUA coatings.

Coating	LED lamp (mW/cm^2^)	Excimer lamp (mW/cm^2^)	UV mercury lamp (mW/cm^2^)	Coating thickness (μm)	Curing time (s)
LED 0	0	30	300	60	10
LED 200	200	30	300	60	10
LED 500	500	30	300	60	10
LED 800	800	30	300	60	10
LED 1000	1000	30	300	60	10
EX 0	500	0	300	60	10
EX 10	500	10	300	60	10
EX 20	500	20	300	60	10
EX 30	500	30	300	60	10
EX 40	500	40	300	60	10
UV 150	500	30	150	60	10
UV 225	500	30	225	60	10
UV 300	500	30	300	60	10
Control	0	0	300	60	10

### Characterization

The FT-IR spectra of coating samples were analyzed by studying KBr pellets in a FTIR spectrometer (Nicolet IS10, Thermo Nicolet Corporation, Madison, Wisconsin, USA). The integration function in Origin software was used to calculate the peak area of the coating samples at 810 cm^−1^ before and after curing. The XPS analyses were performed to identify the functional groups on the testing area of the self-wrinkling coating surface (5 mm × 5 mm × 2 mm) by using an XPS spectrometer with a pass energy of 10 eV and nonmonochromatic MgKα and AlKα X-radiations (ESCALAB 250Xi, Thermo Fisher Scientific, Waltham, USA). The elemental composition and surface contents of the coating samples were analyzed by energy dispersive spectrometry (EDS). The macro-morphology and structure of self-wrinkling samples were observed by SEM (Quanta, FEI, Waltham, USA). The surface roughness of coating samples was characterized by an extended depth-of-field 3D microscope (EDF 3D, VHX-6000, Keyence, Japan). The wavelength, and amplitude of wrinkling coating samples on the coating surface (10 mm × 10 mm × 1 mm) were characterized using atomic force microscopy (AFM) (Dimension Edge, Bruker, USA) by section mode. The Young’s modulus of PUA coatings with different energy intensities were also characterized by AFM tape mode^19,20^. The surface gloss, abrasion resistance, hardness and adhesion test of the coating are carried out according to Chinese standard GB/T 17657-2022. The WCAs of self-wrinkling PUA coatings were measured six times with deionized water (7 μL) at different places by employing a contact angle system (Data Physics, Germany). The average values from three parallel measurements were reported. According to the Chinese standard QB/T1901.2-93, the anti-fingerprint performance of self-wrinkling PUA coatings was measured with artificial sweat^21^. A high-temperature friction and wear testing machine (HT600, Zhongke Kaihua, China) was used to simulate the contact of human hands with the coating surface. A 5 N (0.5 kg) force was applied on the surface of the self-wrinkling coating samples at a speed of 200 r/min for 10 min. The influence of surface topography on the skin-tactile sensitivity of the coating was determined using the friction coefficient^22^.

## Results and discussion

### Surface chemical analysis

**Figure 2:**
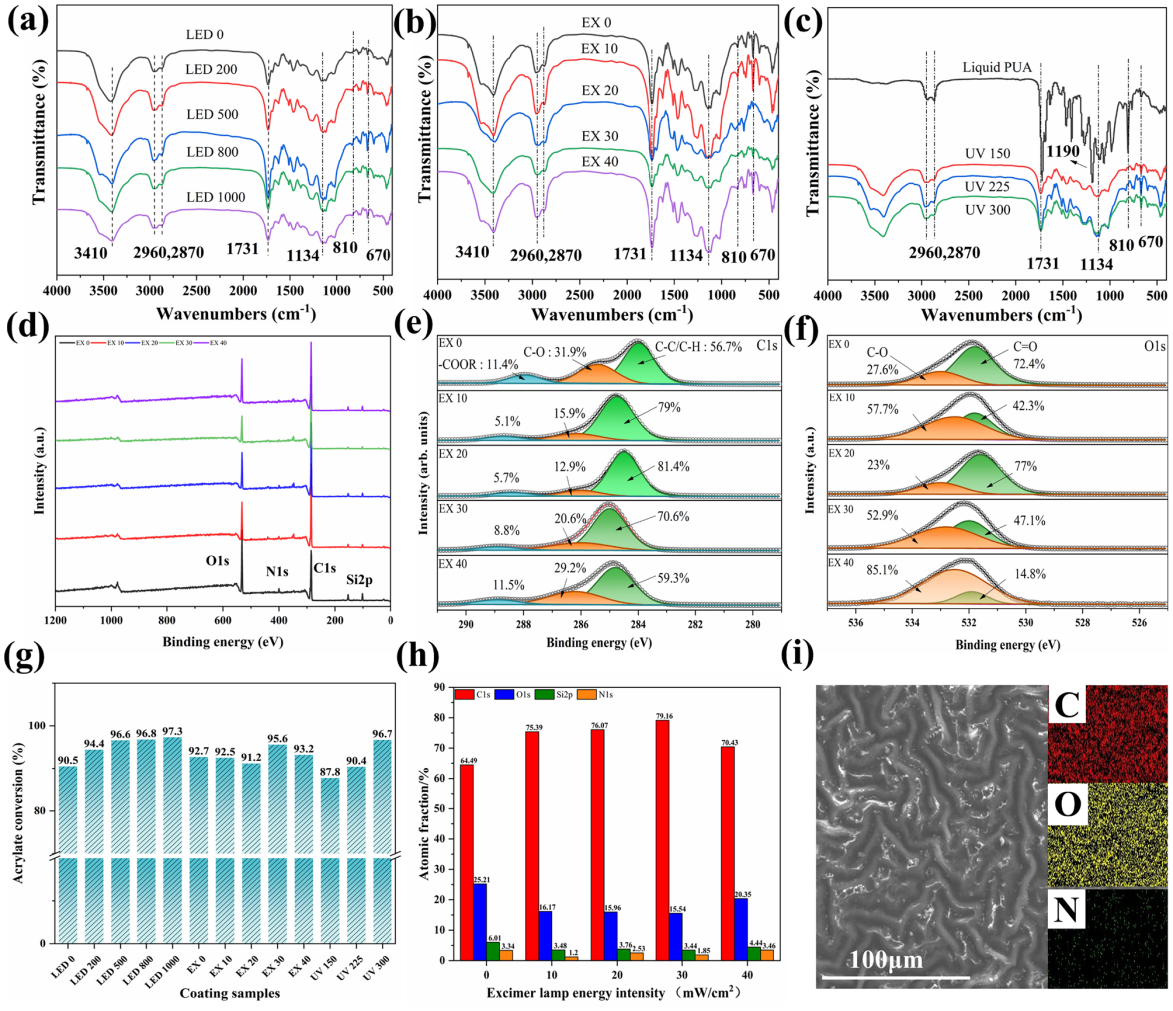
Surface chemical analysis of self-wrinkling PUA coating. (**a–c**) FT-IR spectra of self-wrinkling coating surface for different energy intensities. (**d–f**) XPS spectra, C1s spectrum, and O1s spectrum of self-wrinkling coating samples with different excimer lamp energy intensities. (**g,h**) Acrylate conversion and atomic fraction of coating samples. (**i**) SEM–EDS images of of C, O, N elements on self-wrinkling coating surface at × 100 and × 1.0 k magnification.

Figure 2a–c shows the FTIR spectra of self-wrinkling PUA coatings under the LED, excimer, and mercury lamps at different energy intensities. The bands at 2960, 2870, and 1731 cm^−1^ are the characteristic absorption bands for the PUA coating^23^. The peaks at 2960 and 2870 cm^−1^ are attributed to the stretching vibrations of CH_3_ and –CH_2_– groups, respectively, while the peak at 1731 cm^−1^ represents the stretching vibration of the C=O bond. The C=O bonds are mainly derived from the ester bond generated in PUA, thus further proving the successful preparation of PUA. The strong peak at 3410 cm^−1^ is attributed to the stretching vibration of the N–H bond, which occurs due to the formation of –RCOONH_2_ during polymerization curing^24^. The peak at 1134 cm^−1^ represents the stretching vibration of the C–O–C bond, which occurs due to the production of an ether bond during the curing reaction. The absorption peak at 670 cm^−1^ is generated using residual photoinitiators with benzene rings. Although changes in the energy intensity had little effect on the coating composition, they did lead to significant changes in peak intensity. It is worth noting that compared to cured coatings, uncured coatings exhibit significant absorption peaks at 1190 and 810 cm^−1^. The absorption peak at 1190 cm^−1^ is the unsaturated ester in the coating, and the absorption peak at 810 cm^−1^ is the unreacted C=C bond in the liquid coating. The peak area of the C=C bond is related to the curing degree of the coating^25^. Furthermore, the chemical reactions on the surface of the coating vary slightly when irradiated with different types of ultraviolet light. After pre-curing with LED lamps, the ester bond at the peak at 1731 cm^−1^ was significantly enhanced, indicating the incomplete cross-linking occurred under the irradiation of short-wave LED lamps. When the coating was irradiated with the excimer lamp, the absorption peaks at 1134 and 670 cm^−1^ disappear. The 172 nm excimer produced a highly excited acrylate substance which formed free radicals. The free radicals could then undergo a cross-linking reaction to produce a higher network density on the top layer of the coating^26^. When the mercury lamp was irradiated, the absorption peaks at 1731 and 1134 cm^−1^ are significantly enhanced. Due to the high intensity of the mercury lamp, the coating can be fully cured and cross-linked.

The XPS spectra of the self-wrinkling PUA coating under different energy intensities of excimer lamp are shown in Fig. 2d–f. It has been established that the energy changes of LED and mercury lamps had little effect on the chemical structure of the self-wrinkling coating (Fig. S2). Figure 2d shows that the XPS peaks correspond to C1s and O1s. The nitrogen peaks are also found in the XPS spectra, which correspond to the urethane bond. The C1s XPS spectra shown in Fig. 2e show three peaks; the peaks at 284.8, 286.0, and 288.5 eV correspond to the C–C, C–O, and –COOR bonds, respectively. Compared with the LED 0 coating, the peaks of coating samples cured by the excimer lamp decrease at 286.0 eV and 285.4 eV and increase at 284.8 eV. Meanwhile, the C–C bond content increases from 56.7 to 79% at an excimer lamp energy of 10 mW/cm^2^, while the C–O and COOR contents reduce from 31.9 and 11.4 to 15.9% and 5.1% respectively. At an excimer lamp energy of 20 mW/cm^2^, the C–C bond content becomes the highest (81.4%).This indicates that after the excimer lamp treatment, the C–C bond conversion rate of the coating significantly improves and the content of small molecules (ester and ether) decreases^27,28^. The O1s XPS spectra shown in Fig. 2f also prove this phenomenon. The peak at 532 eV is attributed to the C=O bond in acrylic acid and the peak at 533 eV is attributed to the ester bond in aminomethacrylate. After excimer lamp treatment, the C=O bond content in the coating sample decreases from 72.4 to 42.3% and the C–O bond content increases from 27.6 to 57.7%. This proves that excimer lamp irradiation further improved the curing degree and crosslinking density of the coating. It is worth noting that at an excimer lamp energy of 20 mW/cm^2^, the C–O and C=O bond contents are 23% and 77%, respectively, because the UV funnel effect leads to rapid curing of the coating^29,30^.

The curing degree of self-wrinkling coating samples with different energy intensity is shown in Fig. 2g. The conversion rate of the curing degree of polyurethane acrylate coating was calculated by using Eq. (1):

1 $$\text{Conversion }\left(\text{\%}\right)= \left(1- \frac{{M}{\prime}}{M}\right)\times 100\text{\%},$$

where Mʹ is the peak area of the cured coating at 810 cm^−1^, and M is the peak area of the uncured coating at 810 cm^−1^.

After treatment with different curing energy intensity, the curing degree of the coatings have significant changes. As the energy intensity of LED and mercury lamps increases, the curing degree of the coating continues to increase. When the energy of the LED lamp increases from 0 to 1000 mW/cm^2^, the curing degree of the coating increases from 87.8 to 97.3%. When the energy of the mercury lamp increases from 150 to 300 mW/cm^2^, the curing degree of the coating also increases from 90.5 to 96.7%. However, there is no linear relationship between the curing degree of the coating and the energy of the excimer lamp. When the energy of the excimer lamp is 30 mW/cm^2^, the curing degree of the coating sample reaches 95.6%, which is higher than other samples. This may be due to the formation of dense amplitude wrinkles on the surface of the self-wrinkling coating^31^.

The atomic fraction of self-wrinkling coating samples was shown in Fig. 2h. With an increase in the excimer lamp energy intensity, the C content increases from 64 to over 70% and the O content decreases significantly. The C and O contents of the self-wrinkling coating samples subjected to an energy intensity of 30 mW/cm^2^ are 79.16% and 15.54% respectively, the best curing effect is also observed at this energy intensity. The coating surface and element distribution on self-wrinkling coating surface are shown in Fig. 2i. It can be clearly observed that C and O elements are uniformly distributed on the surface of the coating, indicating that the coating forms a dense structure during the UV curing process. The N element is equally evenly distributed but in small quantities, which is caused by the amide produced during the curing process.

### The regulation of self-wrinkling coatings

**Figure 3:**
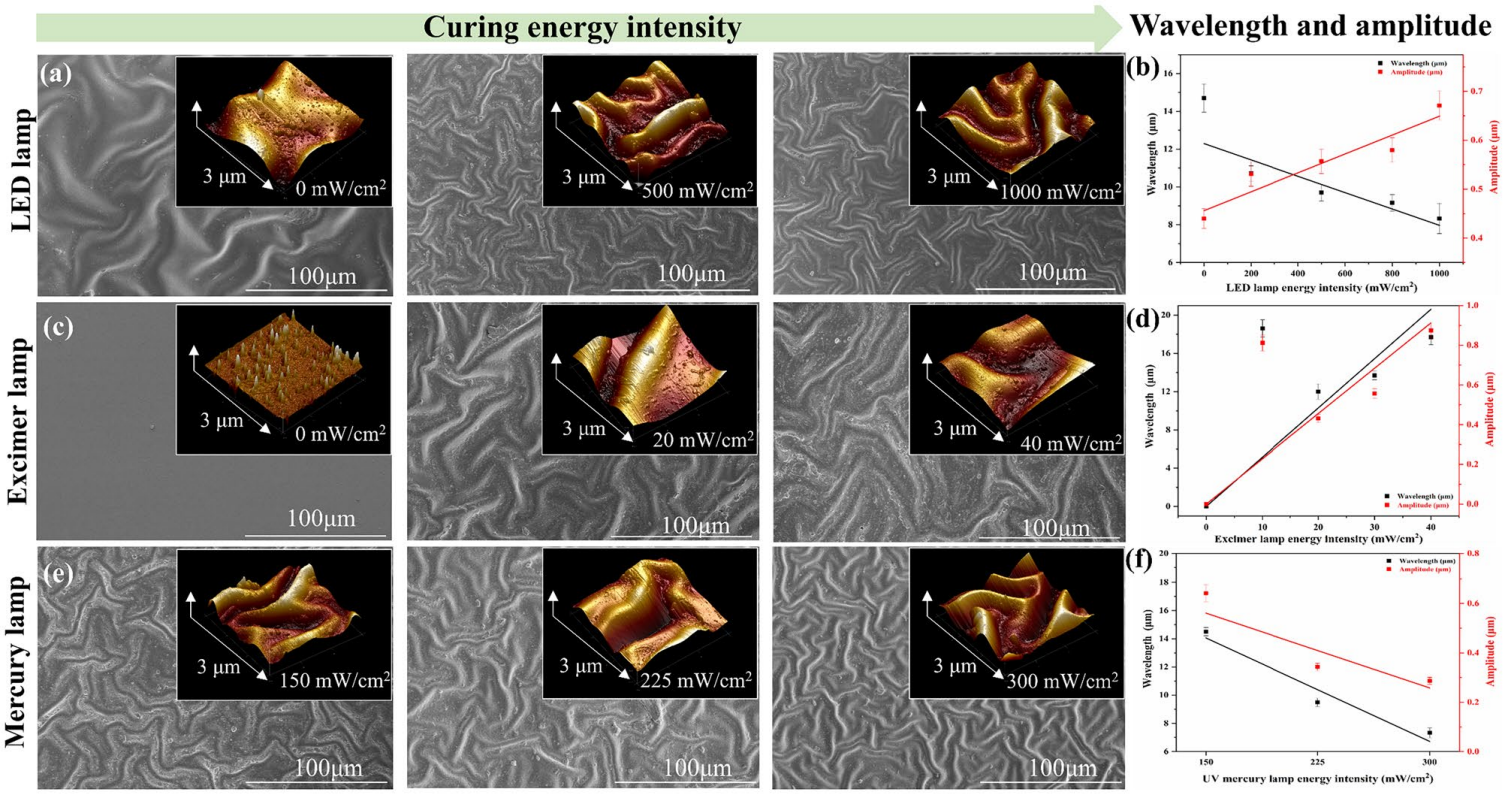
SEM and AFM images of self-wrinkling PUA coatings subjected to different energy intensities. (**a,c,e**) The self-wrinkling PUA coatings exposed to the LED, excimer and mercury lamp with different energy intensities. (**b,d,f**) Average wavelength (λ, black dot and line, left vertical axis) and amplitude (A, red dot and line, right vertical axis) of the self-wrinkling coatings under different energy intensities of the LED, excimer and mercury lamp. Error bars represent the variance of five independent datapoints.

To investigate the factors influencing the preparation of the wrinkled surface, the morphologies, wavelength, and amplitude of PUA self-wrinkling coatings subjected to different energy intensities have been characterized using SEM and AFM, and the results are shown in Fig. 3. The wavelength and amplitude of wrinkles on the coating surfaces are obtained by AFM section mode^32^. As shown in Fig. 3a,b, the energy intensity of LED is negatively correlated with the wavelength sum of the wrinkled structure, but positively correlated with the amplitude. The wavelength and amplitude of the self-wrinkling coating without LED lamp treatment are 14.7 μm and 0.44 μm respectively. With increasing LED lamp energy intensity, the wavelength of the wrinkles decreases from 14.7 to 8.3 μm. Meanwhile, the amplitude increases slowly from 0.44 to 0.670 μm when the energy intensity of the LED lamp is 0–1000 mW/cm^2^. These results indicate that the LED lamp plays an important role in the curing process^33^. In the absence of the LED lamp, molecules in the coating are cured too fast, which produces a large number of long chain molecules that result in a higher internal shrinkage stress, higher wavelength, and lower amplitude on the coating surface. With increasing LED lamp energy intensity, the crosslinking degree of self-wrinkling coating increases. Polymers have a tendency to produce short chain molecules and lower stress, thus the wavelength of the wrinkled structure is decreasing and the amplitude is increasing.

Compared to LED lamps, excimer lamps have a more significant effect on the wavelength and amplitude of surface wrinkles in coatings. Figure 3c,d show the wavelength and amplitude of the self-wrinkling coating exhibit a positive correlation with the excimer lamp energy intensity^34^. In the absence of the excimer lamp, there are no evident wrinkles appear. With the increasing of excimer lamp energy intensity from 0 to 40 mW/cm^2^, the wavelength and amplitude of the self-wrinkling coating is increasing to 17.7 and 0.874 μm. This indicates that a continuous increase in the excimer lamp energy augments the crosslinking depth, which in turn increases the wavelength and amplitude again. Interestingly, at an energy intensity of 10 mW/cm^2^, the wavelength and amplitude of the self-wrinkling coating increase to 18.6 and 0.812 μm respectively. This phenomenon can be a result of the weak penetration ability of the excimer lamp, which formed a thinner hard layer on the coating surface. Figure 3e,f show the self-wrinkling coatings prepared using the UV mercury lamp with different energy intensities of 150, 225, and 300 mW/cm^2^. A control group without UV mercury lamp treatment has not been set up because without this treatment the coating cannot be cured. After the UV mercury lamp energy intensity increases, the wavelength and amplitude of the self-wrinkling coating exhibit a negative correlation. When the UV mercury lamp energy increases from 150 to 300 mW/cm^2^, the wavelength and amplitude of the self-wrinkling coating decrease from 14.5 and 0.742 to 7.33 and 0.287 μm, respectively. The higher irradiation intensity of mercury lamp augments curing speed, which leads to surface wrinkles exhibiting lower wavelengths and amplitudes^35^.

From the above results, it is obvious that the formation of wrinkles is because of the different penetration depths of the three light sources. Typically, the 172 nm excimer lamp has high energy intensity but the lowest penetration depth of 50–100 nm, while the mercury lamp has high energy intensity and high penetration depth of several centimeters, which is suitable for the fully cured of the coating^36,37^. The combined curing of these two light sources is the most fundamental cause of wrinkles generation. In addition, 365 nm LED lamps with low energy intensity and moderate penetration depth are used for pre-curing to reduce the horizontal stresses on the coating surface when wrinkles are generated. This provides us with new ideas for regulating the structure of self-wrinkling surface. When other conditions remain unchanged, the UV penetration depth can be controlled by changing the irradiation intensity during curing^38^. Changing the UV penetration depth can alter the thicknesses of the top and bottom layers, while the wrinkle morphology of the coating surface can be regulated without altering the coating thickness.

### The formation mechanism of self-wrinkling surface

**Figure 4:**
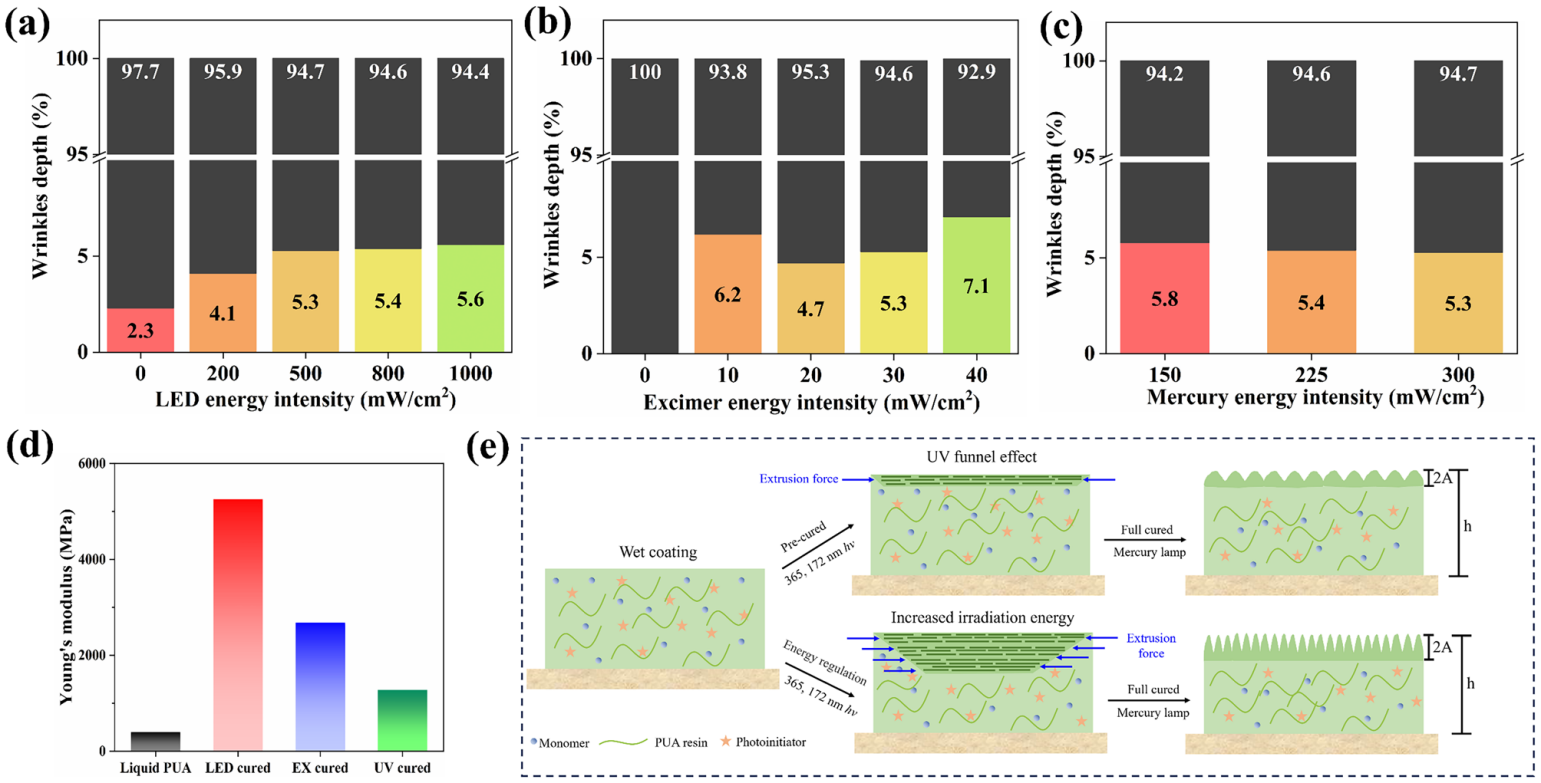
(**a–e**) The wrinkles depth of coating samples with different energy intensity. (**a**) LED lamp, (**b**) Excimer lamp and (**c**) Mercury lamp. The coating total depth is black bar. The wrinkle depth is colour bar. (**d**) The Young’s modulus of coating samples with different lamps. (**e**) The schematic diagram of the regulation of self-wrinkling PUA coating.

To further explore the formation mechanism of self-wrinkling surfaces, the curing depth and wrinkles depth of the coating were tested and shown in Fig. 4a–c and Fig. S3. With the treatment of different energy intensities, the wrinkles depth of coating samples had undergone significant changes. As shown in the schematic in Fig. 1, the amplitude is the half of the total wrinkle depth of in the cross-section of coating. The wrinkles rate of the coating samples was calculated with Eq. (2):

2 $$D\left(\%\right)=\frac{2A}{h},$$

where h is the total depth of coating samples; A is the wrinkle depth of pre-cured; D is the wrinkle depth rate of coating samples.

As shown in Fig. 4a, with the increasing of LED energy in tensity, the wrinkle depth was increased from 2.3 to 5.6%. However, excessive energy intensity could also lead to a decrease in wrinkle depth. Without the treatment of excimer lamps, there were no wrinkles on the surface of the coating, which was consistent with what we observed on the surface. The wrinkle depth of coating samples was increasing from 6.2 to 7.1% with the excimer energy of 10–40 mW/cm^2^ (Fig. 4b). Furthermore, the wrinkles depth was slightly decreased from 5.8 to 5.3% with the increasing of mercury energy intensity (Fig. 4c). The results indicate that the penetration depth of pre-cured increases because of the increasing curing energy of LED and excimer lamps, which is the main reason for the wavelength and amplitude changes of surface wrinkles on the coating.

According to the linear yield theory, the difference in Young’s modulus between the pre-cured layer and the uncured layer is also an important reason for the formation of wrinkles on the wrinkled surface. The Young’s modulus of the coating samples cured by LED lamps, excimer lamps, and mercury lamps respectively is shown in Fig. 4d. The Young’s modulus of liquid PUA was only 400 MPa according to Choi et al.^39^, while the coating samples cured by LED lamp, excimer lamp and mercury lamp were 5254, 2684 and 1279 MPa respectively. This proves our hypothesis that there is a difference in the Young’s modulus between the pre-cured layer and the uncured layer in the coating. The difference promotes the shrinkage of the coating surface, leading to the formation of wrinkled structures.

Figure 4e shows the schematic diagram of the regulation of self-wrinkling PUA coating. The formation of self-wrinkling coatings follows the gradient curing theory^40^. Based on the wrinkled structure of the coating, it can be concluded that curing energy intensity have an impact on the surface morphology of the coating. In the process of photopolymerization, the main forces between the C=C bond and molecular chain in uncured PUA are hydrogen bonding and van der Waals forces^41^. After the coating solidifies, the C=C double bond becomes a C–C single bond, and the reduction of the bond space can cause slight shrinkage of the coating. In the gradient curing theory, UV light irradiation on the surface of the coating produces a light funnel effect, which leads to the formation of a homogeneous and non-stationary structure in the liquid coating^42^. This generates shrinkage stress and extrusion force on the plane direction, which are the main reasons for the self-wrinkling of the coating surface. In conclusion, the increasing energy intensity of LED lamp, excimer lamp, and mercury lamp can regulate the wrinkled morphology of the coating surface. When coating samples prepare without LED lamp treatment, the excimer lamp directly illuminates the coating in a low penetration depth and insufficient extrusion force on the plane. This leads to a larger wavelength and lower amplitude than other samples. With the increase of LED lamp energy intensity, the wrinkled structures on the coating surface continuously increase and form a dense wrinkled surface due to the increase of extrusion force. Furthermore, the excimer lamp plays an important role in the preparation of self-wrinkling coating. Without the treatment of excimer lamps, the shrinkage stress and extrusion force on the coating surface are too weak to form wrinkled surfaces. By increasing the excimer lamp energy intensity, the wavelength and amplitude of wrinkles are significantly increased. Besides, due to the reduction of curing time, the curing speed of the coating samples limits the generation of wrinkled structures, and the wavelength and amplitude of self-wrinkling PUA decrease with the increase of mercury lamp energy intensity.

### Self-matting and physical performance

**Figure 5:**
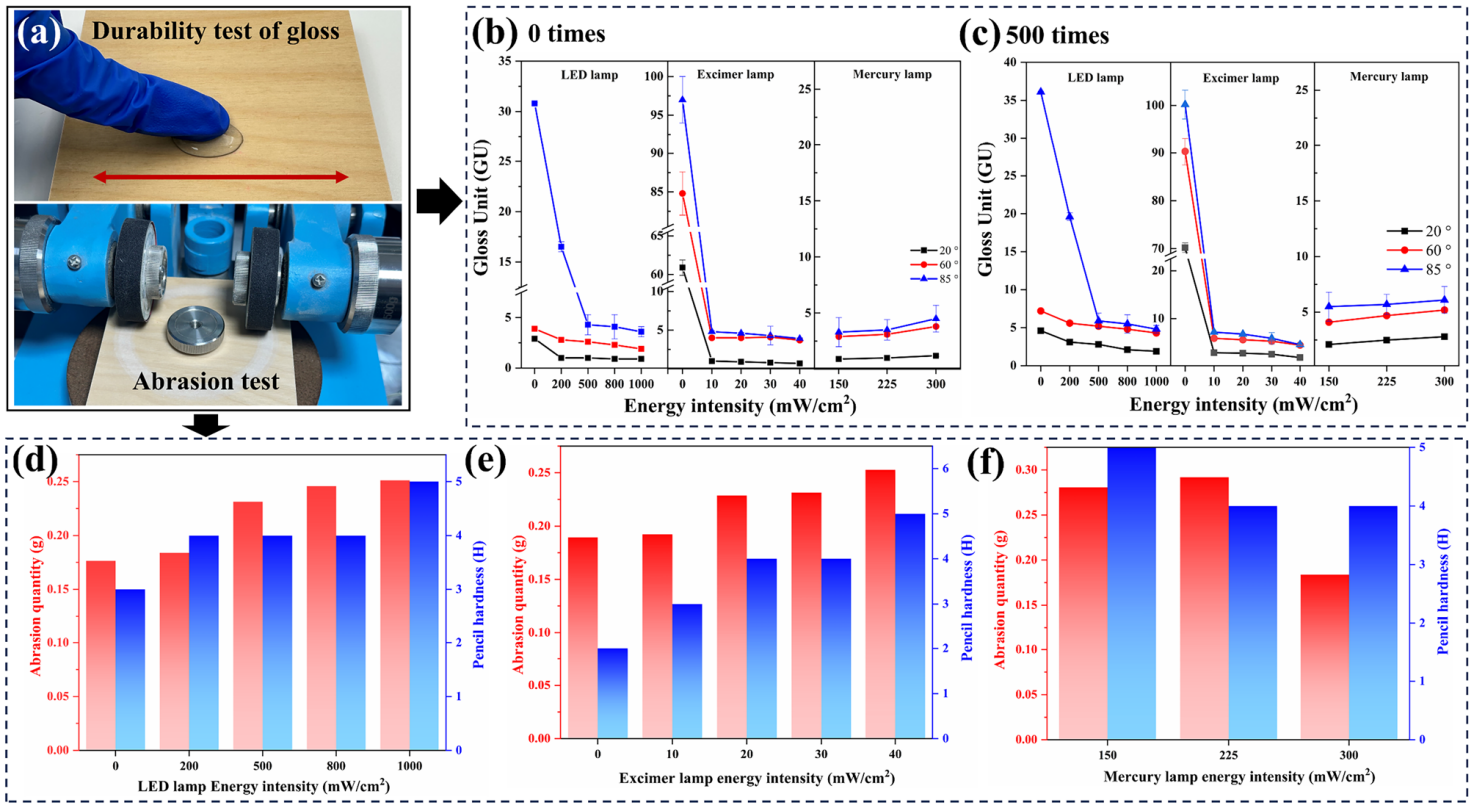
(**a**) Digital images of anti-fingerprint and abrasion tests. (**b,c**) The gloss value of self-wrinkling PUA coating samples after 0 and 500 times of anti-fingerprint test. (**d,e**) The abrasion quantity and hardness of self-wrinkling PUA coating sample surfaces.

Through preliminary exploration and research, we found that the self-wrinkling PUA coating has excellent self-matting and mechanical properties. To further simulate the application scenarios of the self-wrinkling coating, the glossiness, mechanical properties, and thermal properties of coating samples with different energy intensities were tested. The results were shown in Fig. 5. Self-matting and physical performance of self-wrinkling coatings. Based on the method of Wu et al.^35^, we used artificial sweat to rub against the surface of self-wrinkling coatings to verify the durability of coating surfaces, the amount of artificial sweat used is 5ml, and the friction distance is 5 cm. Figure 5a shows the gloss values of the self-wrinkling coatings at 20, 60, and 85° without durability test. The self-matting performance of self-wrinkling coatings is closely related to their wrinkled structure. With the continuous increase of LED energy intensity, the glossiness of the coating surface decreased from 2.9, 3.9, and 30.8 GU to 0.9, 1.9, and 3.6 GU at 20°, 60°, and 85°, respectively. For the coating samples with alteration excimer energy intensity, the glossiness significantly decreases with the increase of wavelength and amplitude of wrinkles^43^. At an excimer lamp energy intensity of 40 mW/cm^2^, the minimum gloss value at 85° is only 0.7 GU. For the changing of mercury energy intensity, the gloss value is higher than other samples due to the decreasing of wrinkled structure. These results are consistent with the trend of changes in wrinkle morphology discussed earlier^44^. Figure 5b shows the gloss value of coating samples after durability test. After 500 times frictions, the gloss value gradually decreases with the wear of the wrinkled structure. At 85 °C, the samples of EX 40 group still have a low glossiness of 4.6GU. The results indicate that the wrinkled structure on the self-wrinkling coating surfaces have good durability.

Figure 5d–f shows that the abrasion quantity and pencil hardness of self-wrinkling coating samples with different energy intensities. As the energy intensity increases, we find that the abrasion quantity of the self-wrinkling coating is negatively correlated with the increase of wrinkled structure, while the hardness is positively correlated with wrinkled structure. The hardness of normal PUA coatings that without the LED/excimer/mercury lamp treatment equals ~ 2H^45^. For the LED groups, the abrasion quantity is increasing from 0.1765 to 0.2511 g/100r with the energy intensities improving, while the hardness is enhanced from 3 to 5H. Similarly, the hardness of excimer lamp groups is also enhanced from 2 to 5H, while the wear resistance of self-wrinkling coating samples is decreasing. Besides, the hardness and abrasion quantity are changing from 5H, 0.2805 g/100r to 4H, 0.1838 g respectively with the increasing of mercury energy intensity. When a vertical force is applied to the self-wrinkling coating surface, the wrinkles protect the coating surface and improve its hardness. However, when a lateral force is applied to the coating surface (abrasion quality test), the coating becomes prone to damage and its wear resistance cannot be improved because the lateral force and wrinkle shrinkage are applied in the same direction^46,47^. Moreover, the thermal decomposition behavior of self-wrinkling coatings has been studied in Fig. S4 and Table S1. A higher energy intensity can increase the degree of curing and reduce curing time, which can effectively increase the crosslinking density and improve thermal stability of the coating. Thus, the prepared self-wrinkling coating surface shows high hardness and low wear resistance.

### Wettability and anti-fingerprint performance

**Figure 6:**
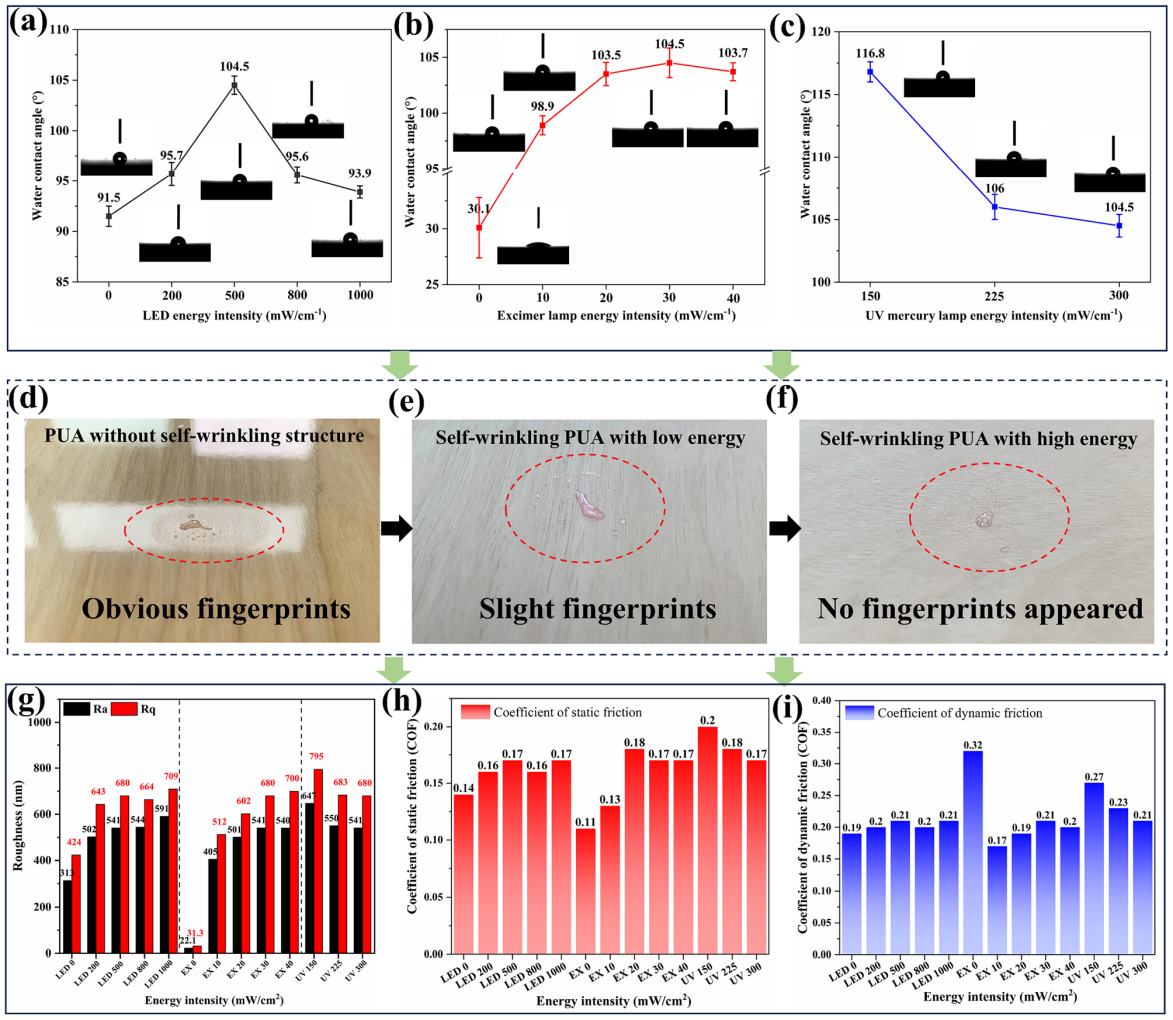
The wettability and anti-fingerprint performance of self-wrinkling coatings. (**a–c**) The WCA of self-wrinkling coating sample surfaces with different energy intensities. (**d,e**) The anti-fingerprint performance of control PUA and self-wrinkling coating samples with different energy intensities. (**g–i**) The Roughness, static friction and dynamic friction coefficients of self-wrinkling coating samples.

After being cured using different lamps, the self-wrinkling coating surface exhibits excellent hydrophobicity, anti-fingerprint properties and tribological performance, the results are shown in Fig. 6. The WCAs of self-wrinkling coating samples subjected to different energy intensities are shown in Fig. 6a–c. Compared to the coating samples without a self-wrinkling surface, the WCA of self-wrinkling coatings tends to increase from 30.1° to 104.5°. With an increase in the LED lamp energy intensity, the WCA of the coating surface is slightly increasing from 91.5° to 104.5°. When the excimer lamp energy intensity is 0–30 mW/cm^2^, the WCA of the coating sample increases significantly from 98.9° to nearly 105°. However, with a further increase in the energy intensity, WCA tends to reduce slightly. Besides, the UV mercury lamp energy intensity reduce the hydrophobicity of self-wrinkling coatings. It is worth noting that the self-wrinkling coating cured by LED/EX/mercury system with 500, 30 and 300 mW/cm^2^, the WCA of coating surfaces ranges between 95° and 105°, which is similar to the WCA of the human skin surface when dry^48^.

Figure 6d–f shows that the anti-fingerprint performances of self-wrinkling coating samples are clearly improved after using the proposed curing system. Compared to PUA coatings without self-wrinkling surface structures (Fig. 6d), the self-wrinkling coating surfaces treated systematically show no obvious fingerprints after the anti-fingerprint test Fig. 6e,f. By regulating the energy intensity, the anti-fingerprint performance of the coating can be altered. When the irradiation intensity of each lamp in the system is increased, the anti-fingerprint performance of the coating surface is significantly enhanced. As shown in Fig. 6f, the surface of self-wrinkling PUA coating appears no fingerprint with high energy intensity.

To investigate the relationship between self-wrinkled pattern and surface skin-tactile sensitivity. Surface energy, roughness and the coefficient of friction (COF) of PUA coating with different energy intensities are measured in Fig. 6h,i. By combining the results of WCAs and Table S2, increasing the curing energy intensity of self-wrinkling coating results in an increase in the surface energy of the coating^49^. The surface energy values of self-wrinkling coating samples treated with the curing system range from 29 to 30 mN/m, which is consistent with the surface energy value of human skin that ranges from 25 to 30 mN/m in a previous study^50^. The WCA and surface energy of this particular self-wrinkling coating (104.5° and 29–31 mN/m, respectively) are close to those of the human skin surface. Figure 6g shows the roughness of self-wrinkling coatings subjected to different energy intensities. After LED and excimer lamp treatment, the Ra and Rq of the coating has been significantly improved. However, the roughness of the mercury lamp decreases with the increase of energy intensity, which may be due to the faster curing rate, resulting in the inability to form wrinkles better. After the PUA coating is cured using the LED, excimer, and mercury lamps at energy intensities of 500, 30, and 300 mW/cm^2^ respectively, the Ra and Rq of self-wrinkling coating are 541 and 680 nm, which is also consistent with the roughness of young children skin in Ohtsuki’s study^51^.

To further investigate the effect of self-wrinkled structure coatings on human tactile sensation, a tribology test has been conducted to simulate the contact of a human hand with the coating surface. The results are shown in Fig. 6h,i, which reveals that the static friction coefficient of the self-wrinkling coating is not high and ranges from 0.1 to 0.2. The static friction coefficient of the self-wrinkling coating subjected to the LED lamp treatment equals ~ 0.16, while its dynamic friction factor increases to a value between 0.19 and 0.21. The dynamic friction coefficient of coatings treated with excimer lamps is significantly lower than that of the coatings not treated using excimer lamps. The PUA coating don’t have wrinkled structure without excimer lamp treatment, which resulting in the the dynamic friction coefficient value is 0.32. According to the experimental results of Highley et al., the dynamic friction coefficient of the human skin surface ranges from 0.2 to 0.3, which is consistent with the friction coefficient of the self-wrinkling coating surface^52^. This result indicates that self-wrinkling coatings with biomimetic self-wrinkled surfaces exhibit a smooth skin-like texture, thus showing significant potential for industrial applications.

## Conclusions

In this research, we synthesized a self-wrinkling PUA coating, which was subjected to curing using a novel LED/excimer/mercury lamp system. After the homogeneous PUA system undergoes UV curing, a self-wrinkling coating is spontaneously formed on the wood surface.

By regulating the curing depth, the differences in modulus between the layers and the shrinkage stresses in the planar direction combine to form the wrinkle structure of the surface. Furthermore, the wavelength and amplitude of wrinkles on the coating surface are controlled by adjusting the irradiation intensity in the curing system. When the PUA coating is cured by the LED, excimer, and mercury lamps with energy intensities of 500, 30, and 300 mW/cm^2^ respectively, the self-wrinkling coating exhibited excellent self-matting, anti-fingerprint and skin-tactile performance. The gloss values of the self-wrinkled PUA coating at 20°, 60°, and 85° equal 1.0, 4.1, and 4.1 GU, thereby satisfying the demands for matting coating. In addition, the hardness and abrasion quantity of the self-wrinkling coating equal 4H and 0.23 g respectively. The WCA and surface energy of coating samples are 104.5° and 29–30 mN/m, which represent significant anti-fingerprint performance on the coating surface. The coefficient of static and dynamic friction are both at the range of 0.15–0.2. Interestingly, these results are consistent with those of the human skin surface. Besides, the wrinkled structure also improves the thermal stability of coating samples. In summary, the self-wrinkling coating exhibits excellent optical properties, anti-fingerprint performance, skin-tactile sensitivity, and physical performance. Thus, the proposed preparation method can be used for large-scale fabrication of self-wrinkling coatings, which in turn have the potential to be applied in wood-based panels, furniture, and leather products.

### Supplementary Information

Supplementary Information.

## Data Availability

Data is provided within the manuscript or supplementary information files. The data that support the findings of this study are available on request from the corresponding author.
