# Supplementary material for: Preparation of a self-matting, anti-fingerprint and skin-tactile wood coating via biomimetic self-wrinkling patterns
Source: Sci Rep. 2024 Jun 10;14:13275. doi: 10.1038/s41598-024-64385-x (PMC11164916; doi:10.1038/s41598-024-64385-x)
Supplement: Supplementary file 1 — Supplementary Information. [file 41598_2024_64385_MOESM1_ESM.docx]

**Supporting Information**

**Preparation of a self-matting, anti-fingerprint and skin-tactile wood coating via biomimetic self-wrinkling patterns**

Yingchun Sun^a^, Ru Liu^a^, Yuhui Sun^a^, Ling Long^a^*

*a.* *Research Institute of Wood Industry, Chinese Academy of Forestry, Beijing 100091, China.*

Yingchun Sun, e-mail: sunyingchun0707@163.com; ORCID: 0000-0003-0120-5545

Ru Liu, e-mail: liuru@criwi.org.cn; 0000-0001-5880-5504

Yuhui Sun, e-mail: sunyuhui@caf.ac.ca;

*Ling long (corresponding author), e-mail: longlingcaf@163.com; ORCID: 0000-0003-1205-9037

**Table of Contents**

**Figure S1…………………………………………………………………3**

**Synthesis of the self-wrinkling PUA coating**

**Figure S2…………………………………………………………………6**

**XPS of self-wrinkling coatings curing by LED and mercury lamps**

**Figure S3…………………………………………………………………7**

**The cross-section SEM images of self-wrinkling coating samples**

**Figure S4…………………………………………………………………8**

**The TGA and DTG curves of self-wrinkling coating samples**

**Table S1.…………………………………………………………………9**

**WCA and surface energy of self-wrinkling coating samples.**

**Table S2………………………………………………………….……10**

**Pyrolysis parameters of self-wrinkling PUA coating samples**

**S1 Methods and characterization**

**S1.1 Preparation of self-wrinkling PUA coating**


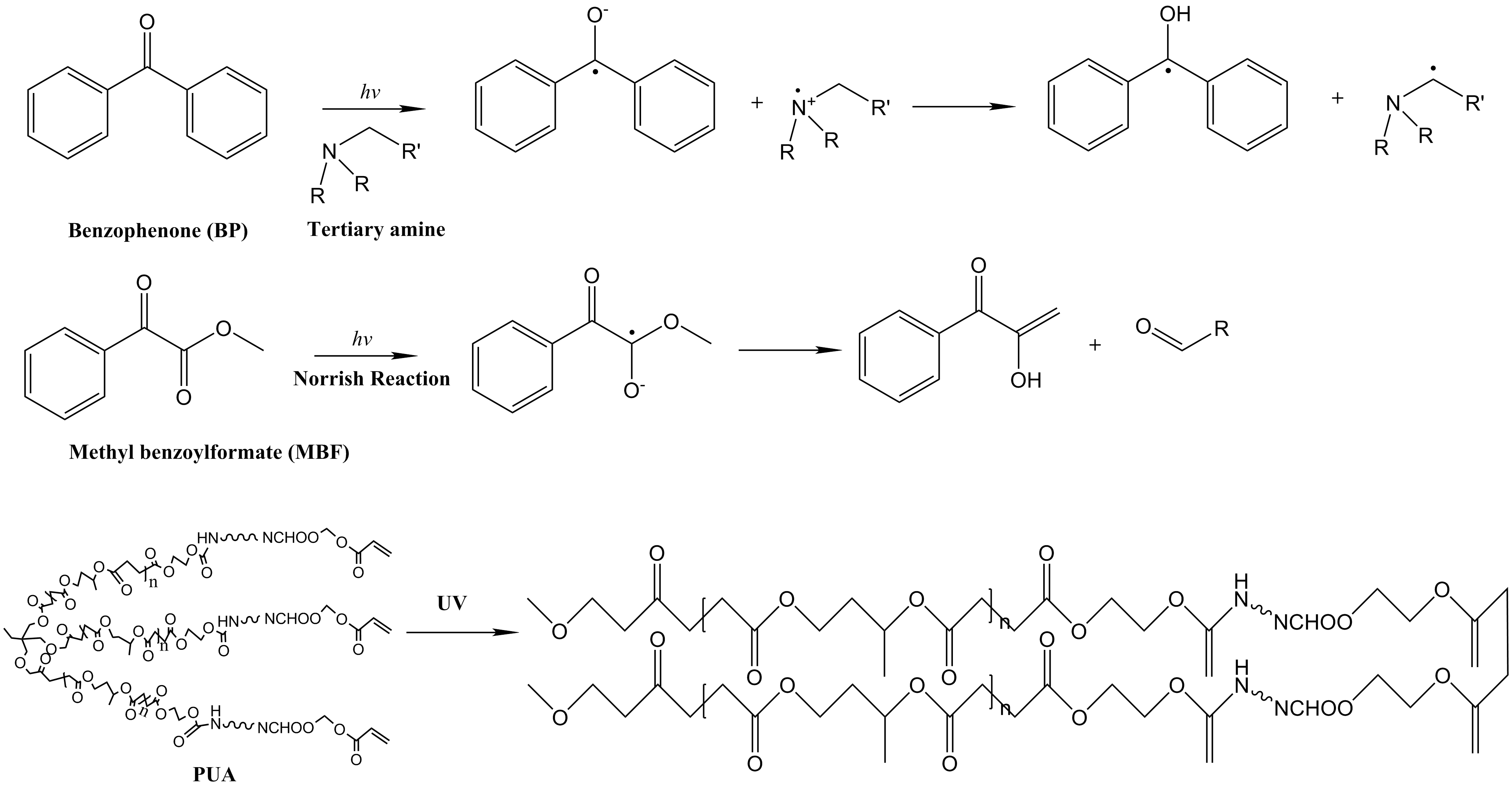


Fig. S1. Synthesis of the self-wrinkling PUA coating

The method used to synthesize the PUA coating (Fig. S1) has been reported in our previous research ^1^, while the synthesis procedure and preparation of the self-wrinkling PUA coating are shown in Fig. 1. Firstly, 100g of PU_3_ and HEA (molar ratio 1:3) were added to a three necked flask and mixed by magnetic stirring at 1000rpm for 30 minutes to obtain the prepolymer. Then, the mixture was heated to 70 ℃ within 30 minutes and reacted for 4h at 70 ℃ to prepare a trifunctional polyurethane acrylate resin. Furthermore, 70g trifunctional PUA resin, 20g TMPTA, and 10g BDDA were added and stirred at 50 ℃ for 30 minutes to obtain UV-PUA prepolymer. Finally, 2g photoinitiator (BP: MBF=1:1) were added in the mixture to prepare the UV-PUA coating.

The mixture of UV-PUA coating was roll-coated to form a thin film on the birch plywood. The Prior to roll coating, the birch plywood was treated with one layer of putty and three layers of UV primer coatings (epoxy primer). The resulting wet coating (60 μm) was exposed to LED lamp, excimer lamp, and UV mercury lamp (PRT-C1103a, PRT-L2 and PRT-1320, Foshan Shunde PURETE Mechanical Co., Ltd.) with different energy intensities for 10 s. In particular, the LED lamp was operated at an energy intensity of 0, 200, 500, 800, and 1000 mW/cm^2^; the excimer lamp was operated at 0, 10, 20, 30, and 40 mW/cm^2^, while the energy intensity of the UV mercury lamp was 150, 225, 300 mW/cm^2^. When the energy intensity of one lamp was changed, it was ensured that the energy intensity of the other two lamps remained constant. The coating samples curing with different energy intensities are defined as LED 0, 200, 500, 800, 1000; EX 0, 10, 20, 30, 40 and UV150, 225, 300. Meanwhile, the control PUA coating samples were cured by mercury lamp at 300 mW/cm^2^ energy intensity. The self-wrinkling coating samples prepared by the 172 nm excimer lamp was protecting in the nitrogen of 99.99% concentration. The preparation details for self-wrinkling PUA coating samples are shown in Table 1.

**S1.2 Characterization**

The gloss values of the cured coating samples were measured using an YG268 gloss meter (3nh, Shenzhen, China) at an incident angle of 20°, 60°, and 85°. The resistance abrasion property was measured by grounding them using a Taber-type abrasion tester (TST-C1020, TST Instruments, Quanzhou, China) with 500 g of taped 180-mesh sandpapers for conducting 100 abrasion revolutions. The hardness of the coatings was tested using a car pencil hardness tester (BY-500g, Pushen, Shanghai, China) at a force of 7.5 kg▪m▪s^-2^. A grade based on the area of the coatings that is detached from the wood substrate was used to determine the adhesion properties, with grade 0 being the best and grade 5 being the worst. A thermogravimetric analyzer (TG) was used to perform thermogravimetric testing on coating samples under nitrogen protection. Test conditions: The nitrogen flow rate is 40mL/min, the temperature range is 30-800℃, and the heating rate is 10℃/min. The stability of the material is determined by the change in mass of the sample during thermogravimetric testing.

The WCAs of self-wrinkling PUA coatings were measured six times with deionized water (7μL) at different places by employing a contact angle system (Data Physics, Germany). The average values from three parallel measurements were reported.

The surface energy of the coating samples was calculated using the Fowkes model ^2^ with equations (1), (2), and (3):

$\sigma_{s}=\sigma_{s}^{D}+\sigma_{s}^{P}$ (1)

$\sqrt{\sigma_{l}^{D}\cdot\sigma_{s}^{D}}+\sqrt{\sigma_{l}^{P}\cdot\sigma_{s}^{P}}=\frac{\sigma_{l\left( 1+\cos\theta\right)}}{2}$ (2)

$\sigma_{s}^{D}=\frac{\sigma_{l\left( 1+\cos\theta\right)}}{4}$ (3)

Where $\sigma_{s}$ is the total surface energy; $\sigma_{s}^{D}$ and $\sigma_{s}^{P}$ are the dispersion and polar components of solid surface energy, respectively; and $\sigma_{l}^{D}$ and $\sigma_{l}^{P}$ are the dispersion and polar components of the liquid surface energy. Accordingly, we tested the WCAs of water and diiodomethane. Based on the facts that $\sigma_{l}^{P}$=51.0, $\sigma_{l}^{D}$=21.8 for water, and $\sigma_{l}$=$\sigma_{l}^{D}$=50.8 for diiodomethane, the surface energy of the coating samples was calculated by substituting the values into equations (1), (2), and (3).

According to the Chinese standard QB/T1901.2-93, the anti-fingerprint performance of self-wrinkling PUA coatings was measured with artificial sweat ^3^. The artificial sweat was consisted by sodium chloride (NaCl, 20g/L), ammonium chloride (NH_4_Cl, 17.5g/L), urea (CH_4_N_2_O, 5g/L), acetic acid (CH_3_COOH, 2.5g/L), lactic acid (C_3_H_6_O_3_, 15g/L) and Rhodamine (1g/L), the PH value of artificial sweat was adjusted to 4.7. Immerse fingers in artificial sweat, use a tissue to absorb excess sweat, and then press your fingers onto the coating surface for more than 5 seconds. Observe the retention of fingerprints on the coating surface after 0 seconds and 30 seconds.

**S2 Results and discussion**

**S2.1 The surface chemical analysis of self-wrinkling PUA coating**

As shown in Fig. S2, the energy changes of LED and mercury lamps had little effect on the chemical structure of the self-wrinkling coating. After curing the coating samples by LED lamp and mercury lamp with different energy intensity, the XPS results of C1s and O1s in the coating surfaces didn’t have obviously changes. The change in C-C, C-O, and - COOR bonds content was less than 5% with the increasing LED energy intenity from 0-1000 mW/cm^2^. With the increasing of mercury energy intensity, the bonds content of the coating surfaces remanined to unchanged.


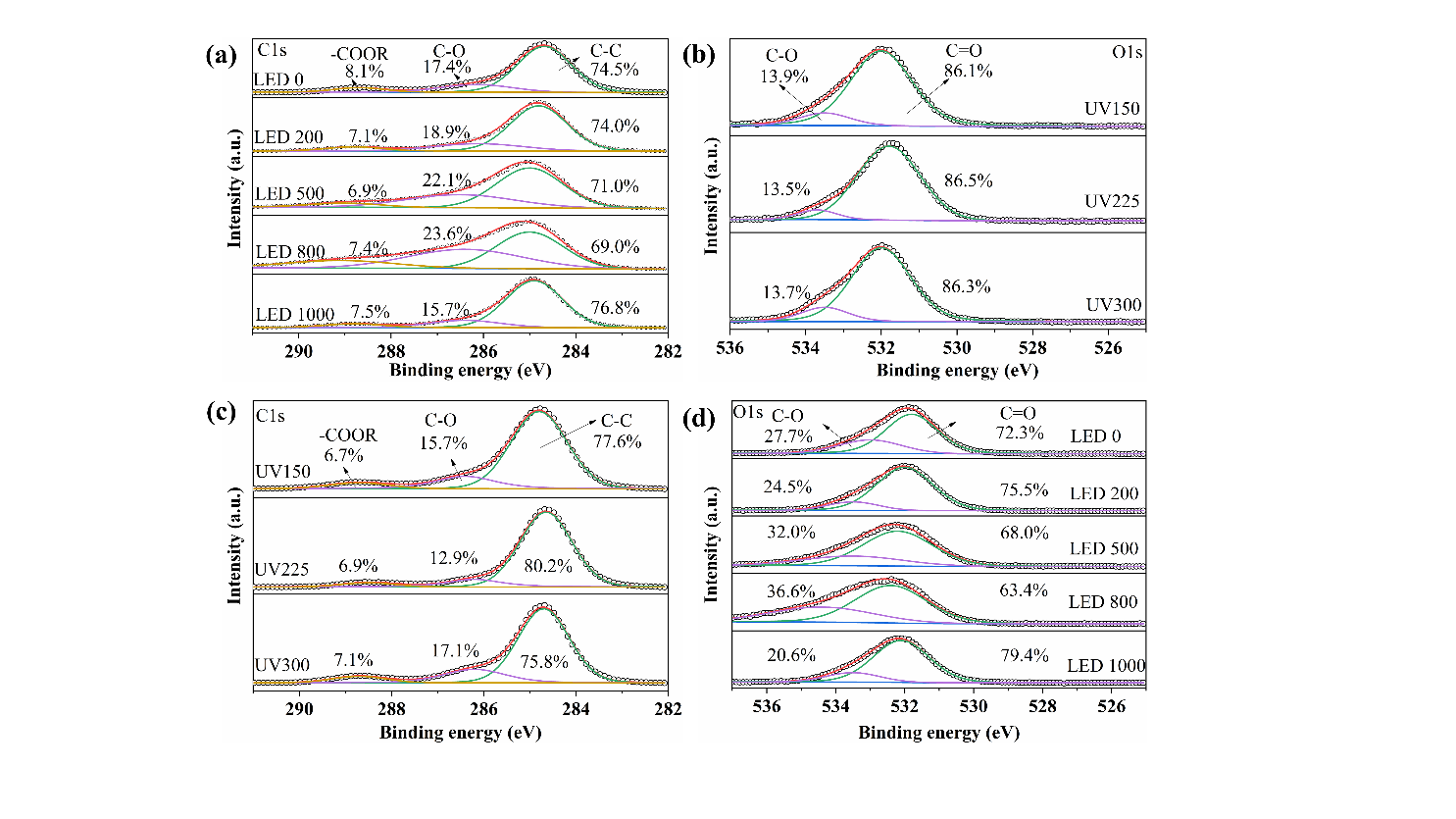


Fig. S2. The self-wrinkling PUA coating with different LED and excimer energy intensity. (a-b) LED energy intensity with 0, 200, 500, 800, 1000 mW/cm^2^. (c-d) mercury energy intensity with 150, 225, 300 mW/cm^2^.

**S2.2 The cross-section images of self-wrinkling PUA coating samples**

To further explore the formation mechanism of self-wrinkling surfaces, the curing depth and wrinkles depth of the coating cross-section were observed by SEM as shown in Fig.S3 (a, b, c). With the treatment of different energy intensities, the wrinkles depth of coating samples had undergone significant changes. With the increasing of LED energy in tensity, the wrinkle depth was increased. However, excessive energy intensity could also lead to a decrease in wrinkle depth. Without the treatment of excimer lamps, there were no wrinkles on the surface of the coating, which was consistent with what we observed on the surface. Furthermore, the wrinkles depth was slightly decreased with the increasing of mercury energy intensity. The results indicate that the penetration depth of pre-cured increases because of the increasing curing energy of LED and excimer lamps, which is the main reason for the wavelength and amplitude changes of surface wrinkles on the coating.


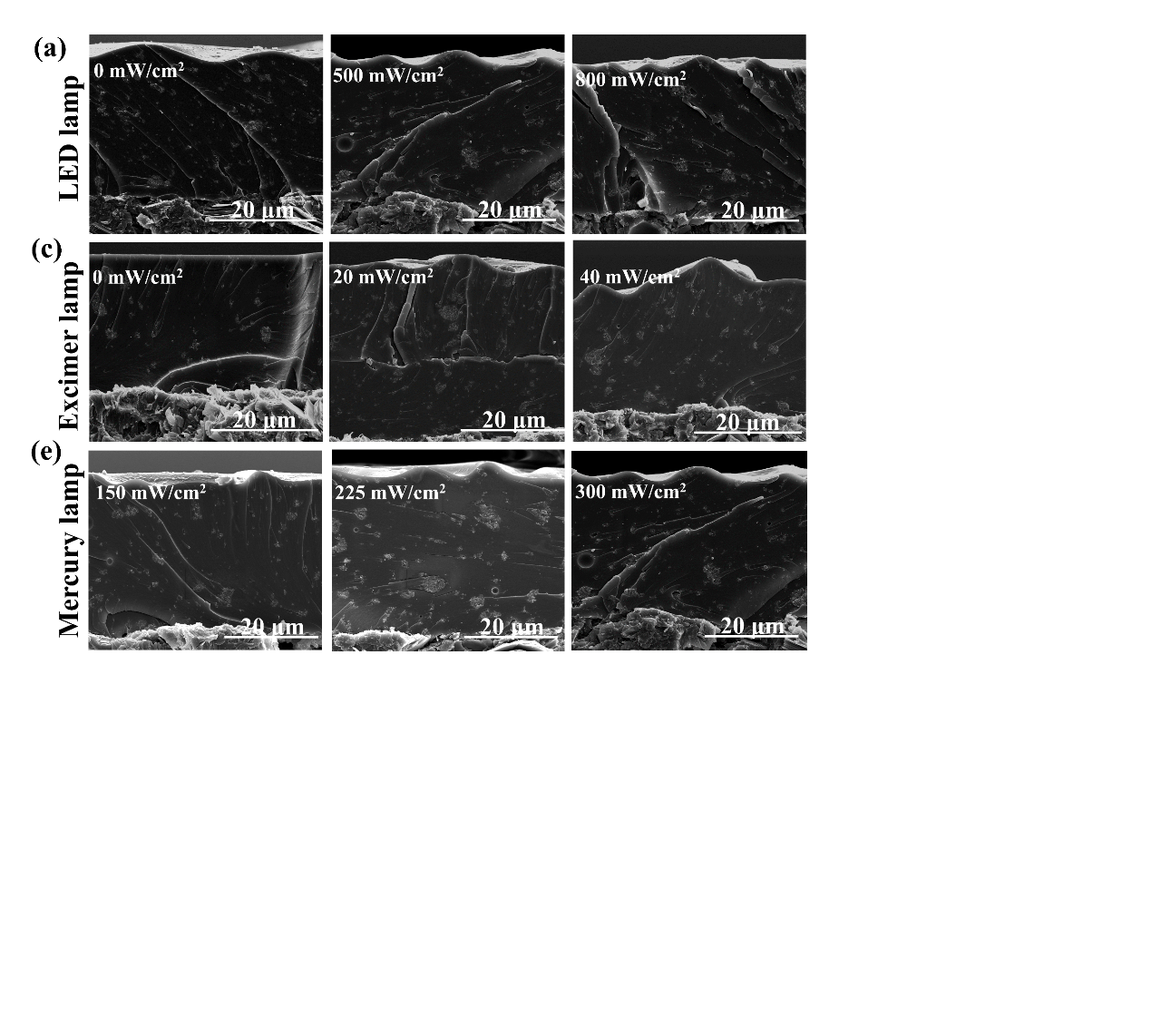


Fig. S3. (a-f) The cross-section SEM images of coating samples with different energy intensity. (a) LED lamp with 0, 500, 800 mW/cm^2^ energy intensities. (b) Excimer lamp with 0, 20, 40 mW/cm^2^ energy intensities. The coating total depth is black bar. The wrinkle depth is colour bar. (c) Mercury lamp with 150, 225, 300 mW/cm^2^ energy intensities.

**S2.3 The TGA and DTG curves of self-wrinkling PUA coating samples**

The effect of energy intensity on the thermal decomposition behavior of self-wrinkling coatings has been studied using a thermogravimetric analyzer, and the results are shown in Fig. S4 (a-c). Meanwhile, to further investigate the pyrolysis performance of the coating samples, pyrolysis parameters are studied (Table S1). Fig. S4 (a) shows that the thermal stability performance of the coating sample improves with a rise in the energy intensity of the LED lamp, while the residual weight rate of the coating samples also increases slightly. When the energy intensity increases from 0 to 800 mW/cm^2^, the residual weight rate rises from 5.39% to 5.72%. When the energy intensity exceeds 800 mW/cm^2^, the residual weight rate begins to decrease. The TGA results of the self-wrinkling coating for different energy intensities of the excimer lamp are shown in Fig. S4 (b). When the energy intensity of the excimer lamp increases from 0 to 40 mW/cm^2^, the residual weight rate first significantly increases and then slowly decreases. The best residual weight rate (16.052%) is observed when the energy intensity of the excimer lamp is 10 mW/cm^2^. Meanwhile, increasing the energy intensity of UV mercury lamps (Fig. S4c) improves the residual weight rate of the coating samples, with the residual weight rates of UV150, UV225, and UV300 samples being 5.30, 5.35, and 5.62%, respectively.

**
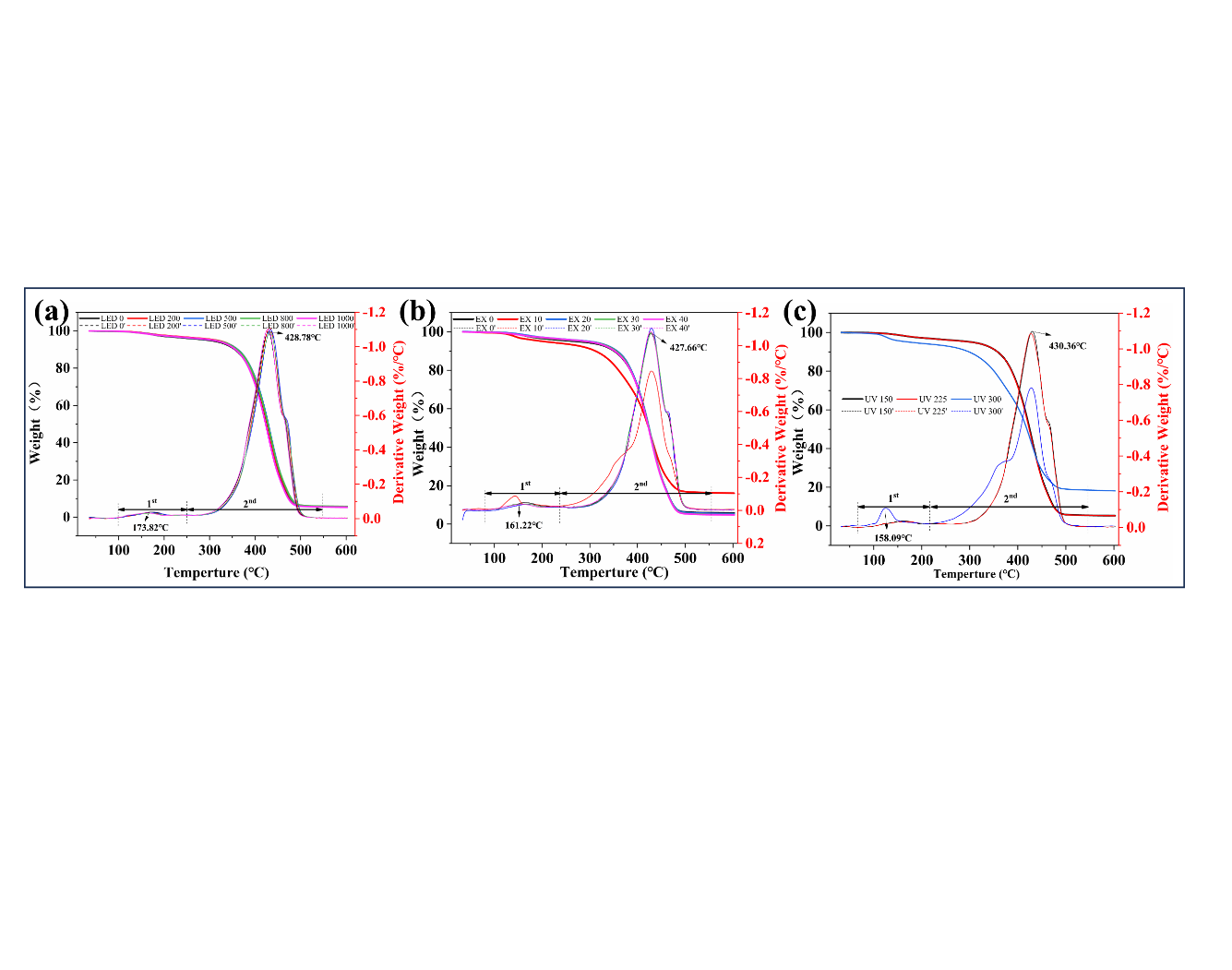
**Fig. S4. The TGA and DTG curves of self-wrinkling PUA coating samples.

The decomposition of all coating samples occurs in two stages ^4^. The peak temperature in the first degradation stage ranges between 150–170 ℃, owing to the C-C bond breakage on acrylate at a weight loss rate of less than 5%. The peak temperature in the second degradation stage is ~430 ℃, owing to the C-O bond breakage on polyurethane at a weight loss rate of 90%. Table S1 shows that the T5% and T10% temperatures of the self-wrinkling coating samples cured with LED, excimer, and mercury lamps were higher than those of the untreated coating samples. Moreover, as the energy increases, the weight loss rates of the first and second degradation stage gradually decrease. This indicates that augmenting the energy intensity can improve the thermal stability of the self-wrinkling coating, which is consistent with our FTIR spectroscopy results ^5^. A higher energy intensity can increase the degree of curing and reduce curing time, which can effectively increase the crosslinking density and improve thermal stability of the coating.

Table S1. Pyrolysis parameters of self-wrinkling PUA coating samples

| Coating | T_5%_  (℃) | T_10%_  (℃) | T_50%_  (℃) | 1st degradation stage | | 2^nd^ degradation stage | | Residue  (%) |
| --- | --- | --- | --- | --- | --- | --- | --- | --- |
|  |  |  |  | WL（%） | DTG_max_(℃) | WL(%) | DTG_max_(℃) |  |
| LED 0 | 298.7 | 355.7 | 423.7 | 3.22 | 173.8 | 91.39 | 428.8 | 5.39 |
| LED 200 | 313.8 | 360.2 | 425.5 | 2.66 | 163.7 | 91.62 | 430.2 | 5.72 |
| LED 500 | 305.8 | 359.2 | 426.1 | 3.08 | 175.1 | 91.30 | 430.9 | 5.62 |
| LED 800 | 307.3 | 358.1 | 425.1 | 2.92 | 162.4 | 91.36 | 430.0 | 5.73 |
| LED 1000 | 310.1 | 358.3 | 424.4 | 3.77 | 162.5 | 90.89 | 429.6 | 5.34 |
| EX 0 | 273.1 | 351.0 | 423.5 | 4.69 | 161.2 | 89.55 | 427.7 | 5.76 |
| EX 10 | 282.8 | 352.1 | 422.1 | 3.83 | 162.0 | 80.11 | 428.0 | 16.05 |
| EX 20 | 298.4 | 355.9 | 423.1 | 4.30 | 168.8 | 90.30 | 428.1 | 5.70 |
| EX 30 | 305.9 | 359.2 | 426.1 | 3.08 | 175.1 | 91.30 | 430.9 | 5.62 |
| EX 40 | 292.6 | 351.1 | 421.7 | 4.17 | 157.2 | 91.08 | 426.4 | 4.70 |
| UV 150 | 304.0 | 357.7 | 424.7 | 4.05 | 158.1 | 90.63 | 430.4 | 5.30 |
| UV 225 | 302.5 | 357.9 | 423.7 | 3.03 | 162.4 | 91.68 | 427.5 | 5.35 |
| UV 300 | 305.9 | 359.2 | 426.1 | 3.08 | 175.1 | 91.30 | 430.9 | 5.62 |

T_5%_, T_10%_, and T_50%_ are the temperatures of coatings that undergo a weight loss of 5%, 10%, and 50%. WL is the weight loss percent of the coating samples. DTG_max_ is the peak temperature in the DTG curve.

The pyrolysis performance of the coating samples, pyrolysis parameters are studied (Table S1). T5%, T10%, and T50% represent the temperatures of coatings that undergo 5%, 10%, and 50% weight loss (WL); WL represents the weight loss percent of the coating samples. Besides, the WL values in the DTG curves for the two degradation stages are also shown in Table S1. This indicates that augmenting the energy intensity can improve the thermal stability of the self-wrinkling coating, which is consistent with our FTIR spectroscopy results. A higher energy intensity can increase the degree of curing and reduce curing time, which can effectively increase the crosslinking density and improve thermal stability of the coating.

**S2.3 WCA and surface energy of self-wrinkling coating samples**

Table S2. WCA and surface energy of self-wrinkling coating samples.

| Coating  samples | Contact angle (°) | | Dispersion component ($\sigma_{s}^{D}$) | Polar component  ($\sigma_{s}^{P}$) | Surface energy  (mN/m) |
| --- | --- | --- | --- | --- | --- |
|  | Water | Diiodomethane |  |  |  |
| LED 0 | 91.5 | 58.9 | 29.2084 | 2.0454 | 31.2538 |
| LED 200 | 95.7 | 58.3 | 29.5537 | 1.0744 | 30.6281 |
| LED 500 | 104.5 | 60.2 | 28.4599 | 0.1109 | 28.5707 |
| LED 800 | 95.6 | 58.3 | 29.5537 | 1.0744 | 30.6281 |
| LED 1000 | 93.9 | 58.3 | 29.5537 | 1.4095 | 30.9632 |
| EX 0 | 30.1 | 22.7 | 40.1033 | 3.4393 | 43.5426 |
| EX 10 | 98.9 | 59.2 | 29.0357 | 0.6033 | 29.6390 |
| EX 20 | 103.5 | 59.8 | 28.3247 | 0.1089 | 28.4336 |
| EX 30 | 104.5 | 60.2 | 28.1134 | 0.1109 | 28.2243 |
| EX 40 | 94.3 | 56.1 | 30.8175 | 1.1781 | 31.9956 |
| UV 150 | 116.8 | 61.4 | 24.1937 | 1.2532 | 25.4469 |
| UV 225 | 106.0 | 60.6 | 29.3588 | 0.5997 | 29.9585 |
| UV300 | 104.5 | 60.2 | 28.4599 | 0.1109 | 28.5707 |

When the LED lamp energy intensity changes, the polarity component of the coating surface increases; however, the total surface energy remains the same. This may be because more free radicals are generated on the surface with an increase in the LED lamp energy intensity, thus forming polar groups and increasing the polarity component. As the energy intensity of the excimer lamp increases, the polarity and dispersion components associated with the surface energy of the coating increase evidently. Since the wavelength and amplitude of the wrinkles increase, the surface roughness of the coating is enhanced along with the hydrophobicity of the self-wrinkling surface. The surface energy of the self-wrinkling coating decreases from 43.5426 to 28.2243 mN/m when treated with an excimer lamp energy intensity of 0–40 mW/cm2. Besides, the surface energy increases after the coating is subjected to the UV mercury lamp treatment. The surface energy values of self-wrinkling coating samples treated with the UV mercury lamp range from 29 to 30 mN/m, which is consistent with the surface energy value of human skin that ranges from 29 to 30 mN/m in a previous study. Thus, the self-wrinkling coating surface has a biomimetic surface structure similar to that of human skin.

**Reference**

1. Sun, Y. *et al.* 33 A novel self-wrinkled polyurethane-acrylate wood coating with self-matting, anti-fingerprint performance and skin-tactile feeling *via* excimer lamp/UV curing. *RSC Adv.* **13**, 7300–7311 (2023).

2. Fowkes, F. M. 36 ATTRACTIVE FORCES AT INTERFACES. *Ind. Eng. Chem.* **56**, 40–52 (1964).

3. Wu, L. Y. L., Ngian, S. K., Chen, Z. & Xuan, D. T. T. 37 Quantitative test method for evaluation of anti-fingerprint property of coated surfaces. *Applied Surface Science* **257**, 2965–2969 (2011).

4. Passauer, L. A case study on the thermal degradation of an acrylate-type polyurethane wood coating using thermogravimetry coupled with evolved gas analysis. *Progress in Organic Coatings* **157**, 106331 (2021).

5. Yu, B. *et al.* UV-Curable Functionalized Graphene Oxide/Polyurethane Acrylate Nanocomposite Coatings with Enhanced Thermal Stability and Mechanical Properties. *Ind. Eng. Chem. Res.* **51**, 14629–14636 (2012).
